# Could Pancreatic Islet Transplantation Outcome Be Impacted by Overestimating Islet Volume? Insights From a Serial Section Study

**DOI:** 10.1155/jdr/8940783

**Published:** 2025-08-06

**Authors:** Praveen Kumar Ravi, Sashikanta Swain, Abhijit Sahu, Sipra Rout, Appakalai N. Balamurugan, Pravash Ranjan Mishra

**Affiliations:** ^1^Department of Anatomy, All India Institute of Medical Sciences, Bhubaneswar, Odisha, India; ^2^Wendy Novak Diabetes Institute, Norton Children's Research Institute, Norton Healthcare, Division of Endocrinology, Department of Pediatrics, Pediatric Research Institute, University of Louisville, Louisville, Kentucky, USA

**Keywords:** ImageJ, islet diameter, islet equivalent, pancreatic islet transplantation, serial section, surgical outcome

## Abstract

**Introduction:** The outcome of pancreatic islet transplantation surgery is influenced by factors like islet volume, purity, and dimensions. Single paraffin section measurement may underestimate islet diameter. Researchers have identified limitations in measuring islet equivalent quantity. This study quantified maximum islet diameter using serial sections and compared it with paraffin sections. We also evaluated actual islet volume and compared it with IEQ based on diameter measurements.

**Materials and Methods:** This study utilized pancreatic tissue from six adult human samples. Serial sections were stained immunohistochemically using anti-synaptophysin antibody. Islets were identified and measured using serial sections to determine their diameter and volume. The maximum average diameter across sections was used to calculate the islet diameter. Islet volume was calculated by summing areas across sections and correcting for section thickness and interval. We compared the calculated IEQ based on the diameter and volume.

**Results:** The study revealed significant discrepancies between pancreatic islet diameter measured from single paraffin sections and those determined from serial sections. The mean sectional diameter was 23.37% smaller than the actual diameter (*p* < 0.0001), with larger islets showing a more significant underestimation. IEQ based on diameter was overestimated by 87.51% compared to IEQ based on actual volume, with large islets contributing significantly to this discrepancy (111.7%).

**Conclusions:** Single paraffin section analyses underestimate islet dimensions, especially for islets > 125* μ*m in diameter. Using conversion factors from this study provides accurate size assessments. To enhance transplantation accuracy, it is essential to use robust size calculations rather than binning. Using the islet diameter tends to overestimate their volume, particularly when the islet index is ≥ 1 (as most islets are larger than 150 *μ*m in diameter). This overestimation increases the risk of unfavorable transplantation outcomes. Thus, IEQ should be adjusted to the upper range when the islet index is ≥ 1, accounting for the potential overestimation of islet volume.

## 1. Introduction

**Figure 1:**
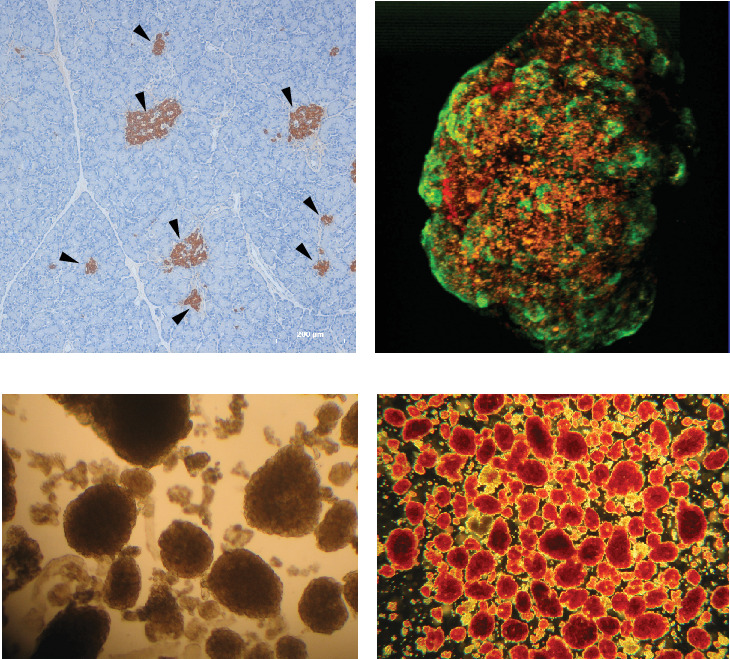
(a) Representative tissue section stained with anti-insulin antibody, demonstrating the presence of islets (black arrowheads) of varying sizes (2D). (b) Magnified 3D view of a single large, isolated islet captured using confocal microscopy, revealing its circularity index of approximately 0.7. (c) Unstained isolated islets with impurities (acinar cells in the background), showcasing the islets of varying sizes under a stereo zoom microscope. (d) Dithizone-stained freshly isolated human islets, demonstrating the purity of the preparation with islets of varying sizes and circularity indices.

The human pancreas exhibits a unique, scattered distribution of islets of varying sizes, shapes, and cellularity, spanning from the head to the tail. The size of islets ranges from a few cells to thousands (Figure 1). Islets collectively constitute approximately 1%–2% of the total pancreatic area, predominantly composed of insulin-secreting beta cells [1]. They are followed by alpha cells, delta cells, epsilon cells, and pancreatic polypeptide cells. Notably, the cellular composition of islets varies with the size of the islets. Recent studies show the importance of the islet dimension in relation to its functionality [2–4]. Larger islets are reported as better functioning in terms of insulin secretion, which may have a protective effect on diabetes [3]. Interestingly, the smaller islets (diameter less than 125 *μ*m) are rich in the beta cell population, predominantly situated in the central core with peripheral nonbeta cells. The larger islets have an intermingled arrangement of relatively fewer beta cells and more alpha cells [5–7]. This intermingled alpha and beta cell arrangement in larger islets allowed the beta cells to respond even to lower blood glucose concentrations [5, 8]. Thus, larger islets with a smaller proportion of beta cells are functionally more active via a paracrine effect than smaller islets with richer beta cells [3]. Increased frequency of larger islets is usually seen in conditions with increased insulin demand like insulin resistance, obesity, pregnancy, and diabetes [9–12]. As age advances, the insulin-secreting capacity of islets decreases, especially in small islets; thus, there is an increase in large islets to tackle the insulin demand [13]. Concurrently, recent studies also report the preferential loss of larger islets predisposing to diabetes mellitus [3]. In contrast, for islet transplantation, smaller islets are preferred for improved outcomes, as larger islets were unable to survive postisolation due to diffusion, whereas smaller islets demonstrated better survival with diffusion [1, 14, 15].

**Figure 2:**
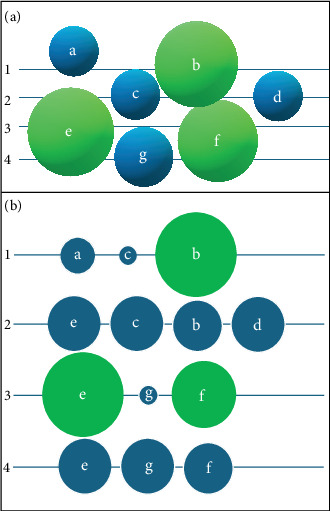
(a) The schematic representation of the islets of varying size (A–G) distributed in the pancreas. Green—large islet; blue—small islet. The transverse lines (1–4) represent the section levels. (b) The appearance of the islets in various tissue sections of (a) (1–4). If the islet diameter is measured using Section 2, leading to an underestimation of the islet diameter of E and B. Similarly, in Section 4, E and F were measured as small islets. Even the size of a small islet is also underestimated, as shown in Sections 1 (A, C) and 3 (G). In a group of seven islets, the chance of getting an islet with its actual maximum diameter is less in all four sections. That implies the degree of underestimation as described.

The complexity of islet architecture and distribution made its quantification process challenging. Since the pancreatic islets are known for high regional variation, random or restricted selection of pancreatic tissue for quantification of islet diameter or proportion will result in multifold under or overestimating islet areas [16, 17]. Furthermore, few studies have selected microscopic fields randomly [18] or only the islet-rich fields for measuring the endocrine proportion, leading to selection bias [16, 17]. Recently, researchers used large-scale computer-assisted methods to analyze the islets to avoid bias due to regional variation in pancreatic islets [4, 16, 17]. However, the single two-dimensional paraffin section may not be sufficient to calculate the islet diameter. The smaller islet that we measure at a particular section might be the terminal region of the larger islet. In other words, the probability of underestimation of islet size continues to exist when the size of the islets is determined from a single paraffin section (Figure 2). However, overestimation is not possible in the single paraffin section for a simple reason that rarely sections would pass precisely through the center of the 3D islet, measuring the maximum diameter. Apart from the challenges in the islet diameter quantification, measuring the proportion of the beta cells in the large and small islets is further complicated.

In clinical islet transplantation, one of the critical parameters influencing success rates is the islet volume, which is often assessed using the islet equivalency (IEQ) measurement [19–21]. IEQ was calculated by assuming all the islets were exactly spherical, which is not the case (Figure 1) [15]. Several studies have highlighted how this spherical assumption can result in significant miscalculations of islet volume, potentially impacting the evaluation of transplant success and subsequent patient care [22–25]. Thus, we further mathematically converted the area (2D) of the islet measured in the serial section into volume (3D) to determine the IEQ as per the volume (IEQ_Volume_) and compared it with the IEQ measured from the diameter of the islet (IEQ_Diameter_). This comparison will provide insight into the actual volume of the islet and IEQ used during the clinical islet transplantation.

To address the issue of islet size underestimation, this study was aimed at quantifying the actual maximum diameter of a group of islets of varying sizes using serial sections. We then compared these measurements with those obtained from individual paraffin sections (from the same set of serial sections) to assess the extent of size underestimation due to the limitations inherent in single-section measurements. Secondly, we also provided insight into the actual volume of the islets (IEQ_Volume_) and the IEQ based on diameter measurements (IEQ_Diameter_) used in islet transplantation.

## 2. Methods

### 2.1. Immunohistochemistry

After obtaining the institutional ethical committee approval, immersion-fixed, paraffin-embedded tissue blocks from the tail region (islet rich region) of the human pancreas (*n* = 6) from the previous studies were utilized [2, 26]. The 4-*μ*m thick serial sections were obtained, up to 48 sections per block. Serial sections were numbered as A1, A2, A3, A4, B1, B2, B3, B4, C1, C2, C3, C4, and so on. All the first and second sections were stained with H&E and anti-synaptophysin, respectively, to look for islet which starts and ends within the serial sections.

### 2.2. Quantification of Islet Diameter

**Figure 3:**
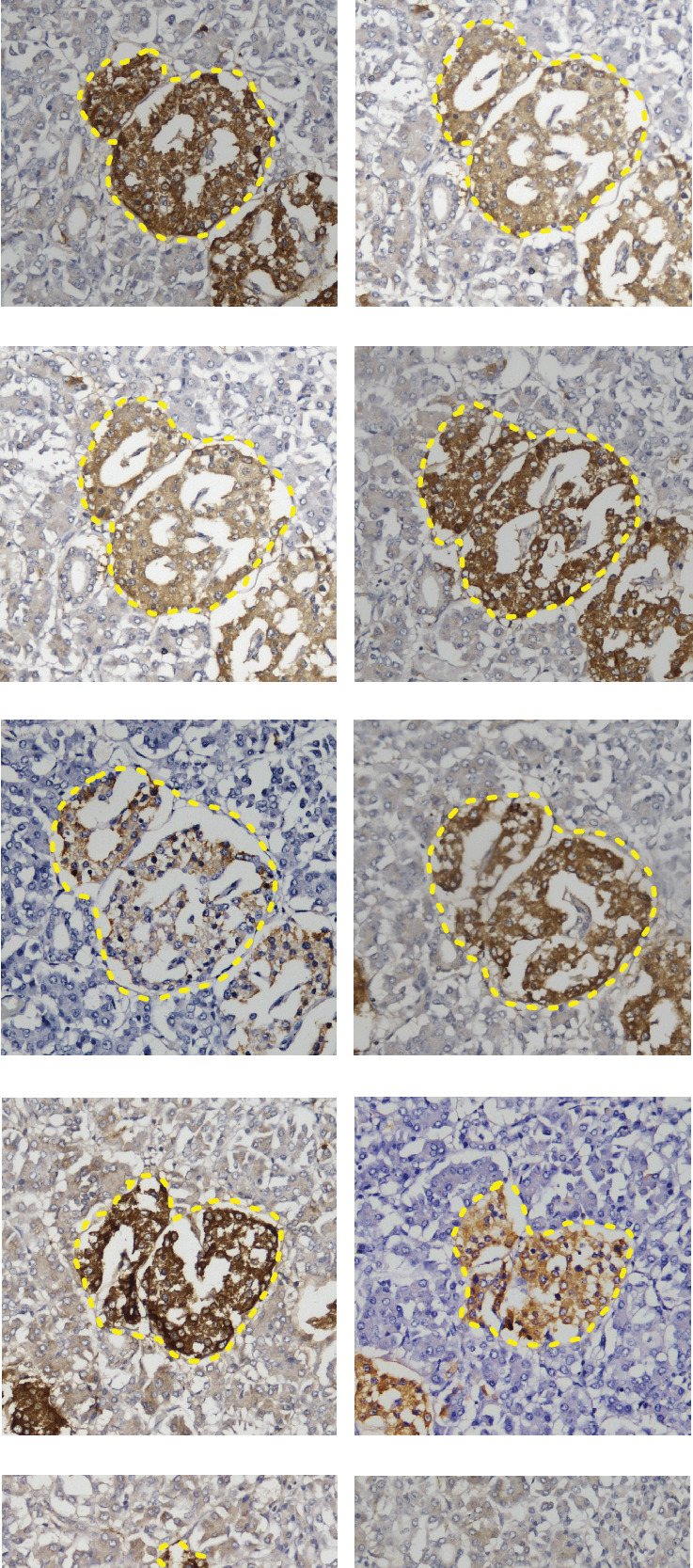
Photomicrograph of consecutive serial sections of a single large islet (20X magnification). This photomicrograph depicts the entire islet from (a) to (l). The islet exhibits a gradual increase in size at the beginning and subsequent decrease. It is evident that islets are not exclusively specific in these serial sections.

We identified islets that begin and end within serial sections by screening through all available sections (Figure 3). Each islet was assessed based on its largest sectional diameter observed across these sections, representing its actual diameter. Specifically, for each section where an islet was present, we calculated the average diameter from the two diameters measured right-angled to each other. Across all sections, the actual diameter (*D*_actual_) of each islet is determined as the maximum average diameter observed:

 $$\begin{array}{c} D_{\text{actual}}=\max\;\left(\frac{D_{1a}+D_{1b}}{2},\frac{D_{2a}+D_{2b}}{2},\cdots \cdots ,\frac{D_{na}+D_{nb}}{2}\right) \end{array}$$

where *D*_*ia*_ and *D*_*ib*_ are the two diameters measured at section *i* and *n* is the total number of sections containing the islet. This method ensures that we capture the largest average diameter of each islet across its sections. The mean sectional diameter (*D*_section_) of each complete islet measured in the particular section was compared with the mean actual islet diameter (*D*_actual_) of the islet present in the particular section to quantify the proportion of underestimation of the islet.

### 2.3. Quantification of Islet Volume

The volume of the islet was calculated using the formula below, without assuming that the islets are perfectly spherical:

 $$\begin{array}{c} V_{\text{islet}}=A_{\text{sum}}\times T\times \text{I} \end{array}$$

where *V*_islet_ is the islet volume of the single islet, *A*_sum_ is the sum of total islet areas from all serial sections of the single islet, *T* is the thickness of the tissue section (in microns) (i.e., 4 *μ*m), and *I* is the interval between examined sections (i.e., 4—every fourth section is analyzed).

### 2.4. Quantification of Islet Equivalent Based on the Diameter (IEQ_Diameter_)

Based on Ricordi et al. methods, IEQ_Diameter_ was calculated using the actual islet diameter measured by the serial section. This assumes that the islets are perfectly spherical.

 $$\begin{array}{c} {\text{IEQ}}_{\text{Diameter}}=\left[\left(n_{50-100}\times 0.167\right)+\left(n_{101-150}\times 0.648\right)+\left(n_{151-200}\times 1.685\right)\right] \end{array}$$

where *n*_50−100_ is the number of islets in the diameter range 50–100 *μ*m, *n*_101−150_ is the number of islets in the diameter range 101–150 *μ*m, and *n*_151−200_ is the number of islets in the diameter range 151–200 *μ*m.

### 2.5. Quantification of Islet Equivalent Based on the Volume (IEQ_Volume_)

IEQ_Volume_ based on the individual islet volume (*V*_islet_) measured in the serial section was calculated by the sum of all the islet volume (*V*_Total_) divided by 17.67 × 10^5^ *μ*^3^ (the volume of a spherical islet with 150 *μ*m diameter, *V*_IE_):

 $$\begin{array}{c} {\text{IEQ}}_{\text{Volume}}=\frac{V_{\text{Total}}}{V_{\text{IE}}} \end{array}$$

where IEQ_Volume_ is the islet equivalent based on the islet volume, *V*_Total_ is the sum of the volume of all islets [∑(*V*_islet_)]  with diameter > 50* μ*m, and *V*_IE_ is the volume of one standard islet equivalent, which is the volume of a sphere with a 150 *μ* diameter. *V*_IE_ = 17.67 × 10^5^  *μ*^3^.

### 2.6. Statistical Analysis

The data were summarized and expressed in mean ± SD. The mean islet diameter and beta cell proportion of the small and large islets were analyzed using a paired *t*-test. *p* value < 0.05 is considered significant. The statistical analysis was performed using the SPSS Version 25.

## 3. Results

### 3.1. Variations in the Sectional and Actual Islet Diameter

**Figure 4:**
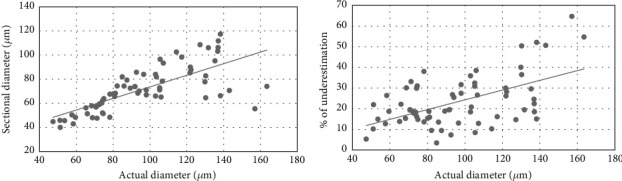
Scatter plot showing the (a) relation of the actual islet diameter with sectional islet diameter and (b) percentage of underestimation with the actual (maximum) islet diameter.

The islet diameter measured using a single paraffin section was significantly smaller than the actual diameter of the islet measured using the serial section. The mean sectional and actual diameter of the islet are 71.79 ± 18.79 and 96.11 ± 28.33* μ*m, respectively. The sectional islet diameter was 23.37% ± 12.35% underestimated compared to the actual diameter measured using the serial section (*p* value < 0.0001). The mean section diameter of the islets was found to be directly proportional to the actual diameter of the islets (*r* = 0.725, *p* < 0.0001) (Figure 4a). Similarly, the percentage of islet size underestimation was also directly proportional to the actual diameter of the islets (*r* = 0.541, *p* < 0.0001) (Figure 4b). Thus, the chance of underestimation is higher when the size of the islet is larger than 125 *μ*m. In other words, when there is a greater number of large islets, the percentage of underestimation of islet size is higher. The mean sectional and actual diameter of the small and large islets was tabulated (Table 1) which confirms the above statement. The underestimation of the islet diameter of large islets is 15% more than that of small islets (*p* = 0.006).

This percentage of underestimation is converted into a multiplication factor for converting the single paraffin section diameter to the actual diameter. Multiplication factors were derived by adding 1 to each underestimation percentage divided by 100 [27]. The multiplication factor formula (conversion factor) for the small and large islets is as follows:

$$\begin{array}{cc} \; & \text{Actual diameter }\left(\text{small islet}\right)=\text{sectional diameter }\left(\text{if sectional diameter is}<100\;\mu \text{m}\right)\times 1.2024,\\ \text{Actual diameter }\left(\text{large islet}\right)=\text{sectional diameter }\left(\text{if sectional diameter is}>100\;\mu \text{m}\right)\times 1.3523. \end{array}$$

### 3.2. Islet Volume Measurement From the Serial Section

The mean islet volume of all the islets measured in the study was 4.65 × 10^5^ *μ*^3^. The mean islet volume of the small and large islets is 2.77 × 10^5^ *μ*^3^ and 12.05 × 10^5^ *μ*^3^, respectively. On average, the volume of a large islet is five to six times more than that of a small islet.

### 3.3. Islet Equivalent Measured by Islet Diameter Versus Actual Islet Volume

IEQ measured using diameter and volume is 57.72 and 30.79 IEQ, respectively. IEQ measured using diameter is overestimated (87.51%) compared to that of IEQ measured by the actual volume of the islet (Table 2). Out of this overestimation, a significant portion is contributed by the large islet (> 125 *μ*m) (Table 2). Thus, the odds of overestimation of IEQ significantly rise when the islet index is ≥ 1 (as most islets are larger than 150 *μ*m in diameter), compared to islet index < 1 (predominantly islets are less than150 *μ*m in diameter).

## 4. Discussion

Pancreatic islets (~1%–2%) are scattered into the ocean of exocrine acini, making it challenging for researchers to quantify. The nature of the distribution of the pancreatic islet is heterogeneous; thus, examining the selected region of the pancreas or selected field of the stained slides leads to quantification bias [18, 28, 29]. Such random selection of tissue or field results in ~2-fold over or underestimation of the results [16, 17]. Further, few studies selected islet-rich regions, which leads to ~5–10-fold overestimation [16, 17]. In recent decades, several studies have employed large-scale, computer-assisted whole slide image (WSI) analysis to address this issue of heterogeneity [4, 16, 17, 30]. WSI capturing and analysis require sophisticated equipment to capture and store. A large-sized image requires approximately 1.5 GB per image when a 1 × 1 cm tissue section is captured at 10X magnification. Due to the practical difficulties, studies that analyzed WSI are done in a single paraffin section, not based on serial sections. Since the islets are scattered heterogeneously, examining a single paraffin section is insufficient to determine the actual diameter of an islet, which might only represent the smaller peripheral region of the larger islet.

The present study determined that the underestimation of the islet diameter in a single paraffin section was 20%–35% depending on the size of the islet. The amount of underestimation was relatively higher when the islet size was larger than 125 *μ*m in diameter. Large islets often appear in multiple serial sections, with only one section potentially passing through the center of the islet, leading to frequent underestimation of their size. Analyzing the serial section of WSIs with large-scale analysis is scarcely possible. Therefore, this conversion factor enables researchers to determine the actual islet diameter from the sectional diameter without needing to perform serial sectioning.

In clinical islet transplantation, IEQ measured using islet diameter was a widely accepted mode of volume calculation. IEQ was first described by Ricordi et al. in 1990 to quantify the volume of the islet transplanted in animal experiments or during clinical islet transplantation [31]. Following that, IEQ was adapted as a standard parameter to quantify the islet volume, due to its relatively easy method of quantification. Islets need to be binned based on the size into 50-*μ*m increments (not required to calculate the exact size), which makes it simpler to measure. The binned islet is then mathematically converted into a number of islets equal to the islet size of 150 *μ*m in diameter, which is the IEQ.

In the Edmonton protocol, the amount of IEQ/kilogram transplanted is considered a crucial factor that determines the success rate. A larger proportion of the IEQ will be contributed by the smaller number of the large-sized islet. In 2010, Kissler et al. conducted a multicentric study to validate the methodology of the quantification of the isolated islets and reported an overestimation of IEQ up to 50% [24, 32]. The intratechnician variation ranges up to 43% [24, 32]. Pisania et al. reported around 90% overestimation of islet volume in IEQ measurement when compared with the nuclei counting method [24, 33]. The huge variation is because of the subjective nature of the islet binning and assuming the islet is perfectly spherical. The studies reported the circularity index of the islets ranging from 0.6 to 0.8, which are ellipsoidal (Figure 1) [24, 25]. In particular, large islets are predominantly ellipsoidal which supports the finding of the present study [25]. Several other studies have documented the poor reliability and validity of the IEQ measurement [23, 24].

The present study documented the overestimation of the islet volume when the IEQ is measured using diameter. The large-sized islet (> 125 *μ*m in diameter) causes a higher discrepancy in IEQ of up to 111.7% when compared to the small islet (59.76%). Thus, the presence of a higher proportion of large islets leads to a multifold overestimation of IEQ, which might result in a reduction of the actual islet volume transplanted, leading to transplant failure.

Studies reported poor transplant outcomes when there is an increased proportion of large islet [15, 34–36] compared to small islets. The unfavorable outcome of the transplantation with a higher proportion of large-sized islets may be attributed to the underestimation of the islet volume during the IEQ estimation process, resulting in a lower islet volume transplanted. The IEQ measurement using the islet binning was easy to calculate in the late 1990s; now, with the advanced imaging and automated computed-based platforms, precise individual islet diameters can be measured to minimize the calculation error of IEQ.

The study's limitations include the exclusive selection of tissue blocks from the tail region of the pancreas. This approach was implemented to enhance the representation of islets within the limited field of view, as the tail region is known for its high islet density. Furthermore, the study utilizes 48 serial sections, enabling the inclusion of islets with a maximum diameter of 192 *μ*m. Islets exceeding this size were excluded from the analysis in both IEQ_Volume_ and IEQ_Diameter_. It is important to note that the diameter and IEQ (calculated based on volume and diameter) were determined solely for islets belonging to the same set. Consequently, the results obtained are not influenced by restricted number of sections. Islet function based on its diameter was not accessed in this study; instead, we discussed based on the published literature. Further clinical/preclinical studies involving isolated islets are necessary to validate the findings of this study.

## 5. Conclusion

In conclusion, islet diameters were consistently underestimated in single paraffin section analyses, especially for islets larger than 125 *μ*m in diameter. By applying the conversion factor determined in this study, researchers can accurately calculate these measurements, thereby overcoming the issue of underestimation and obtaining a more precise assessment of islet size. Secondly, to optimize the accuracy of islet transplantation, it is crucial to move beyond simple binning methods and focus on robust calculations for determining exact islet size. When islets exceed 125 *μ*m in diameter, using the islet diameter alone tends to overestimate their volume, particularly when the islet index is ≥ 1. This overestimation increases the risk of unfavorable transplantation outcomes. Therefore, to mitigate the risk of underestimation, IEQ should be adjusted to the upper range when the islet index is ≥ 1, accounting for the potential overestimation of islet volume.

## Figures and Tables

**Table 1:** Comparison of actual and sectional islet dimension and rate of underestimation. All the compared values are statistically significant. ^@^*p* < 0.0001; ⁣^∗∗^*p* < 0.0001; ⁣^∗^*p* < 0.0001; ^#^−*p* < 0.0001.

**Parameter**	**Large islet (> 125 *μ*m)**	**Small islet (< 125 *μ*m)**	**All islet**
Actual islet diameter (*μ*m)	138.26 ± 10.33^@^	84.98 ± 19.69^∗∗^	96.11 ± 28.33^#^
Sectional islet diameter (*μ*m)	88.59 ± 20.57^@^	67.35 ± 15.69^∗∗^	71.79 ± 18.79^#^
Percentage of underestimation (%)	35.23 ± 16.85^∗^	20.24 ± 8.64^∗^	23.37 ± 12.35

**Table 2:** Comparison of IE_Volume_ and IE_Diameter_ of the small and large islets.

	**I** **E** **Q**_**V** **o** **l** **u** **m** **e**_	**I** **E** **Q**_**D** **i** **a** **m** **e** **t** **e** **r**_	**Overestimation (%)**
Small islets (< 125 *μ*m)	14.41 IE	23.03 IE	59.76%
Large islets (> 125 *μ*m)	16.38 IE	34.69 IE	111.7%
All islets	30.79 IE	57.72 IE	87.51%

## Data Availability

The original data utilized in this study can be obtained from the corresponding authors upon reasonable request.
